# Patch Transporter: Incentivized, Decentralized Software Patch System for WSN and IoT Environments

**DOI:** 10.3390/s18020574

**Published:** 2018-02-13

**Authors:** JongHyup Lee

**Affiliations:** Department of Mathematical Finance, Gachon University, Seongnam-si 13120, Korea; jonghyup@gachon.ac.kr; Tel.: +82-31-750-5255

**Keywords:** software update, decentralized system

## Abstract

In the complicated settings of WSN (Wireless Sensor Networks) and IoT (Internet of Things) environments, keeping a number of heterogeneous devices updated is a challenging job, especially with respect to effectively discovering target devices and rapidly delivering the software updates. In this paper, we convert the traditional software update process to a distributed service. We set an incentive system for faithfully transporting the patches to the recipient devices. The incentive system motivates independent, self-interested transporters for helping the devices to be updated. To ensure the system correctly operates, we employ the blockchain system that enforces the commitment in a decentralized manner. We also present a detailed specification for the proposed protocol and validate it by model checking and simulations for correctness.

## 1. Introduction

Recently, Wireless Sensor Networks (WSN) and Internet of Things (IoT) show noteworthy technical advances and rapid increase in the number of installed devices. The WSN and IoT devices are power-constrained but, basically, they are small computers. Thus, for management and security reasons, they need to be regularly updated. Manufacturers and service providers deliver software updates called ‘patches’, to the devices (from a tiny binary modification to an entire firmware). Despite the importance of timely applying patches to the devices, it is notably difficult to keep a large number of devices in an up-to-date state [1] by two problems: first, Over-The-Air (OTA) delivery of software updates to a number of nodes via multi-hop wireless channels is painful. The performance of the OTA update is quickly degraded as the number of nodes increases [2]. In particular, the lossy channels of wireless communication with varying link quality make it harder to achieve reliable delivery [3]. Second, the attack surface including all the relaying nodes and recipients is overly large to protect all.

To overcome the two problems, there have been efforts on software updates in the WSN and IoT environments. Deluge [4] focuses on the effective OTA delivery. It reliably transfers the software updates via wireless channels. This reliable delivery of software update is extended by mobile sources (initiators) in [5] and by distributed dissemination in [6]. Focusing on the second problem, Lee et al. [7] recently presented CodeDog in-network monitoring of software update delivery with semantic fingerprints of payload. Cheng et al. in [8] proposed the traffic-aware patching to select optimal relaying node to update in order to minimize the propagation of attacks. Additionally, several open source projects are being developed for software updates. Two projects, swupdate [9] and RAUC [10], provide a software update for embedded systems and mainly focus on reliable, fail-safe installation. On the other side, Mender [11] and Resinhup [12] present server and client for the OTA update.

However, due to the large population and the variety of services in the WSN and IoT environment, discovering targets and delivering patches are still challenging. To effectively maintain the software update service, a dedicate management server is required. It should be highly resilient to the power and network loss, and be able to keep track of a list of every device [13]. However, for small manufacturers, it is hard and costly to maintain such highly available and efficient update services. Additionally, a WSN/IoT device consists of software components from multiple providers. If we need to urgently update a commonly-used component, e.g., openssl, the targets would be a number of devices from the multiple vendors.

The difficulty in managing a software update service causes real-world problems. Recent studies show that a huge number of devices are still working without any decent protection from cyber attacks [14]. The out-dated (or never-updated) devices become potential threats. The damage from the cyber attacks by compromised WSN and IoT devices, such as Mirai Botnet [15], is tremendously increasing. Attackers quickly target the devices, having weak login credentials or known vulnerabilities, by rapid searching and scanning (e.g., via Shodan) and perform massive attacks [16]. However, more than 200,000 devices in 2017 still needed the security patch for the Heartbleed attacks that had occurred in 2014 [17]. Furthermore, the software update process itself also needs to be carefully managed. In August 2017, mistakenly updating mismatched devices caused the malfunctioning of hundreds of IoT smart locks used for the Airbnb service [18].

Therefore, for high availability of software update and rapid patch delivery, we apply a distributed service approach to the software update process. In particular, we are ironically inspired by the efficiency of attackers, who are self-interested and distributed. In the distributed service for software update, an independent worker, called a ‘transporter,’ fetches the software patch and delivers it to a proper device. The transporter earns a fee if the patch is acceptable. This distributed approach for software update has benefits in availability and efficiency. It is also beneficial to every participant. Since a device pays the fee for the first delivery of patch, we can encourage the rapid delivery of patches by competing transporters. The providers do not need to maintain a costly management server and the customers can safely use the device in the up-to-date form.

To realize this distributed approach, a solid reward system should be guaranteed for supporting fair exchange of the incentive and the patch. The reward systems on distributed workers have been evolved as Internet services, e.g., Amazon Mechanical Turk [19]. The fair exchange, which is traditionally called as the ‘delivery vs. payment’ problem, is handled by complicated legal prose or escrow services of the help of a mediator [20,21]. When a buyer and a seller cannot agree on finalizing an exchange, the mediator investigates the dispute and select a winner, who will get the fund. Recently, the cryptocurrency systems based on blockchain, Bitcoin and Ethereum, are used to implement the reliable mediator [22,23] in a distributed manner. The blockchain system helps the fair exchange to be automated and trustworthy, since the smart contract on top of blockchain can be working as an enforcer of the predefined rules for fair exchange.

However, building a solid reward system using blockchain still has difficult tasks. Several researchers [24,25,26,27,28] are already reporting the security concerns in the blockchain system. In particular, the attacks on the smart contracts [27] can incur the huge economic damage as we could see in the DAO Hack [29], and Parity Multisig Wallet Hack [30]. Thus, the reward system should protect itself from the blockchain-specific attacks. Furthermore, to prevent possible vulnerabilities in implementing the patch delivering system on blockchain, the reward system should be designed against the realistic security concerns in the context of blockchain. However, the previous fair exchange methods [22,23] do not consider the attacks employing the low-level vulnerabilities in smart contracts, e.g., mishandled exceptions, gas consumption, and fabricated opcodes [27,28].

To sum up, the distributed software update service can enable scalable software update for WSN and IoT, but it can be realized only if a practically secure and fair reward system supports the process. Therefore, in this paper, we propose PatchTransporter, a distributed, incentivized patch delivery approach for the WSN and IoT environments. We design a distributed software update service based on the solid reward architecture with blockchain entities. Moreover, to ensure the fairness for every participant, each participant is protected by self-checking processes, such as the receipt and package validation. We define the roles of *providers*, *transporters*, and *recipients* in delivering patches. A provider prepares encrypted patch packages for devices. A self-interested transporter gathers the patch packages and finds the target devices for delivery. Once the package is successfully delivered, the recipient device creates a smart contract as a *receipt*, which contains the delivery fee for transporter and its redemption condition. If the transporter can also validate the receipt, it reveals the final decryption key, which can unlock the patch payload, to claim the fee. We define three properties to reliably achieve the goals of all participants. To keep the properties, we also draw possible domain-specific attacks on the blockchain reward system for delivering patches. Through model checking and simulation, we check that the proposed system successfully satisfies the properties and prevents the attacks.

This paper makes the following contributions:

1. We define the properties for the distributed software update services and draw realistic domain-specific vulnerabilities for blockchain reward system.
2. We propose an incentivized, distributed patch delivery system, which guarantees the fair exchange and is secure against the blockchain related attacks.
3. We check the correctness of the proposed system and the safety against the attacks through model checking and simulations.

## 2. Blockchain and Smart Contracts

### 2.1. Blockchain

A blockchain is introduced as the infrastructure of Bitcoin [31] to enable a distributed, verifiable ledger. At first, Bitcoin utilizes blockchain only for building its transaction structures on the top of it, but now the use of blockchain is not limited to the cryptocurrency systems. A wide range of industries are actively accepting blockchain to improve their systems from financial institutes [32] and notarizing documents to automating payments and organization. In particular, Bitcoin [31] and Ethereum [33] are representative cryptocurrency systems built on top of the blockchain. Thus, we assume on both systems in the following explanations on blockchain.

*Address and transaction*. The basic elements of blockchain system are *address* and *transaction*. The address represents an account, which stores the money (cryptocurrency). When creating an address, a user generates a public key and a private key pair. The public key is transformed into an address and the private key is used to sign transactions originated from the new address. Thus, only the owner of the private key can create a transaction that transfers the money in the address to other addresses. A transaction basically represents the movement of the money from its inputs to its outputs. Conceptually, the input and the output can be treated like addresses, but, more specifically, the output generally is a condition on an address and the condition means that whoever can prove the ownership of the address can use this money. Thus, the output is connected to another input of the following transaction that proves the ownership, e.g., signing by the private key. That is, every transaction should be authenticated. Hence, the balance of an address is the sum of available outputs, which is called ‘Unspent Transaction Output (UTXO)’.

*Block and blockchain*. The blockchain system consists of distributed nodes. Each node is basically synchronized via peer-txfo-peer communications. When a transaction is published to a node, it is propagated to other nodes. A node that fully participates in the blockchain system called a ‘miner’. The miner node validates all the transactions and builds a ’*block*’ from the newly validated transactions. A block consists of the transactions, the hash of the previous block, and a proof of its validity, which is used in consensus algorithms. The details are different depending on the type and implementation of blockchain. However, the blockchain systems of permissionless participation and the Proof of Work (PoW) consensus algorithm are still dominant. Since the participation as a miner (or a validator) to the blockchain needs no permission, the consensus algorithm of blockchain provides a way for participants to agree on the same fact without trusting each other. Bitcoin and Ethereum use the PoW algorithm with incentives. The blockchain system pays the incentive by giving a right to mint a certain amount of new coins to the miner who finishes a block. However, whenever a miner tries to make a block, she is encouraged to validate the unconfirmed transactions, which are not included in any block yet, and she must solve a cryptographic puzzle, e.g., finding a pre-image of a hash satisfying a certain condition. Due to the security of cryptographic operations, the solution for the puzzle is purely probabilistic. Thus, the miner needs to repeat the computation to make a block until any one in the network succeeds to make a block. The time for a blockchain network to generate a block is called as ‘blocktime.’ Each blockchain system has different target blocktime, and the difficulty of the puzzle is periodically adjusted to maintain the blocktime close to its target. Since a newly generated transaction is validated when it is included in the block, the validation result of the transaction is also delayed for a blocktime in general. In addition, since a block contains a hash value of the latest block, the block proves the existence and integrity of the previous block. This is also true in the relationship between the previous block and the previous block of the previous block. Thus, it makes a chain of blocks, called ‘blockchain’, where a block cumulatively confirms the integrity of the predecessor blocks. If two or more blocks are accidentally created based on the same (latest) block, miners select any of them as its latest block. Since the PoW-consensus algorithm depends on unpredictable computation, the decision on the latest block is probabilistic. Hence, it is infeasible that an ordinary miner intentionally creates a block with forged transactions and sequentially creates another following block on it because the rest of miners (or computing power) reject the invalid block. To sum up, every transaction is confirmed in blocks by miners and the integrity of blocks are guaranteed by the blockchain, i.e., the chain of blocks. The incentives encourage the miners to comply with the system, and the consensus system provides fairness and protects from the attacks to violate the rules among the participants even without any trust. Readers can refer to [34] on underlying mechanisms for building the blockchain system.

### 2.2. Smart Contracts

Besides the addresses and transactions, the *smart contract* adds programming constructs to the blockchain systems. In general, a smart contract is a program code that can be executed in the blockchain and triggered by transactions. Thus, recently, the similarity between the smart contracts and the traditional distributed objects was explored in [35]. Each blockchain system has different smart contract models. In Bitcoin, the smart contract is implemented as a script at the input and output of a transaction. Basically, most Bitcoin transactions use conventional scripts to show the validity of the new transaction, and, more specifically, to prove the ownership of the private key corresponding to the target UTXO [36]. However, by adding more conditions to the script with leveraging other parameters, e.g., locktime, the Bitcoin transaction can handle more sophisticated behaviors [37]. On the other hand, Ethereum has a more generalized smart contract model than Bitcoin. The smart contracts in Ethereum are executed on top of the Ethereum Virtual Machine (EVM), which resides in every miner. In Ethereum, a smart contract code is programmed in the domain-specific programming languages, such as Solidity [38], Vyper [39], Serpent [40], etc., and compiled to an EVM bytecode. The EVM bytecode is deployed to the blockchain via a transaction. If both the deployment transaction and the initiation of a smart contract are successful, the smart contract has an independent address to communicate with. Since it is also an independent account in the Ethereum network, it can have its own money, i.e., Ether, and storage. In addition, it can send and receive transactions with other accounts. Once an instance of smart contract code is deployed, the execution is triggered by transactions. In the Solidity model, one can call a certain function in the smart contract by indicating the exact signature of the function. If a transaction indicates no or incompatible function, the fallback function in the smart contract handles the request.   

*Gas*. The another functional improvement on the smart contract in Ethereum is supporting loops, which is limited in the Bitcoin, thus the smart contract model of Ethereum becomes Turing complete. In the validation of the transactions with smart contracts, every mining node executes the smart contract code on its EVM under the shared Ethereum state. If a smart contract incurs excessive or erroneous execution, all the miners and a whole network can get damage from it. Hence, in Ethereum, every EVM instruction has its own cost, called ’gas’ and the calling transaction should provide enough money (gas) in Ether to execute all necessary instructions (the accumulated gas cost for the execution is multiplied by the flexible gas price). Please refer to [41] for the detail explanations on the smart contract model of Ethereum.

*Security*. The security of the smart contracts are based on the security of the blockchain system. Due to the characteristics of the underlying blockchain, the execution of the smart contract is irreversible like transactions, and the code cannot be modified once it is deployed. However, since the smart contract is executed in the context of blockchain, the unexpected bugs in the smart contract code cause unintentional execution. The bugs are mostly related to the Ethereum-specific execution model, e.g., fallback functions or gas cost. The attacks on the smart contract have been researched in [27,28]. Therefore, when building a secure service leveraging smart contract, one should also manage the security concerns in smart contracts. In particular, we consider the attacks applicable to our approach: intentional incomplete execution, and the excessive gas consumption in Section 3.

### 2.3. Blockchain and IoT

The fundamental challenges in realizing secure services with WSN/IoT devices are related to the restricted performance of the devices. In order to lower the cost, the WSN/IoT devices have low-powered processing units and no security co-processors, such as the Trusted Platform Module (TPM) [42]. Thus, the only limited use of resource-expensive cryptographic operations is allowed. Furthermore, since the WSN/IoT devices may be battery-powered and have long-sleep cycles, the protocol with a number of message exchanges is not suitable.

As one of the practical solutions, the WSN/IoT network externalizes security features, and the blockchain is well matched for the purpose [43,44]. In particular, the blockchain-based services for WSN/IoT is advantageous to solve the conventional challenges of IoT security, such as capacity constraints, deficient architecture, unavailability of services, manipulation susceptibility, etc., [45]. In particular, the smart contract helps the WSN/IoT devices to delegate the security functions by deploying a smart contract as an autonomous agent in the blockchain [43].

From the perspective of performance, overall cost can be reduced by applying the blockchain system to the WSN and IoT environments. A WSN/IoT device joins to the blockchain system as a client (lightweight node), which does not participate in the mining process. Thus, the computational requirement is limited to the operations for creating a transaction or a smart contract, and a single digital signing operation is dominant in energy consumption for both. Once a deputy smart contract is deployed, the WSN/IoT device can let the subsequent messages be authenticated by the blockchain system (miners) as transactions. On the other hand, the blockchain systems using IoT devices as verifiers are also being presented, e.g., IOTA (https://iota.org).

However, the blockchain cannot be the panacea for WSN/IoT. The security service based on the blockchain should be carefully designed under the following considerations:

1. The tolerant latency of the service should be larger than the confirmation time (a blocktime). As we mentioned in Section 2.1, a new transaction needs to wait for entering into a block in order to prove its validity. Thus, the authentication based on the transaction also takes a blocktime at least.
2. The sensitive information can be unintentionally revealed. The blockchain achieves security by the security-through-transparency model. Every transaction, including smart contracts, is shown to all the nodes. In order to preserve privacy of transactions in public blockchain system, sophisticated cryptographic operations, e.g., zero knowledge proofs, are required [46], but it causes too heavy of a load for WSN/IoT devices.

Our approach is designed regarding the limitations of the blockchain system with the WSN/IoT environments. Rapid delivery of software patches is important, but the time scale of software patch is not seconds but hours or days. The blocktime of Bitcoin and Ethereum is 10 min and 15 s, respectively, thus the confirmation times are tolerable enough to use them in implementing secure software patch services. In addition, to prevent unintended information leakage, our approach uses the pre-image of hash values in smart contracts and validates the smart contract before sending sensitive information to protect secrets via misguided transactions, which is explained in Section 3. Finally, the WSN/IoT device in our approach creates only a single blockchain transaction for a successful delivery, which has been tolerable in recent IoT devices [47].

## 3. Distributed Software Update Service

**Figure 1:**
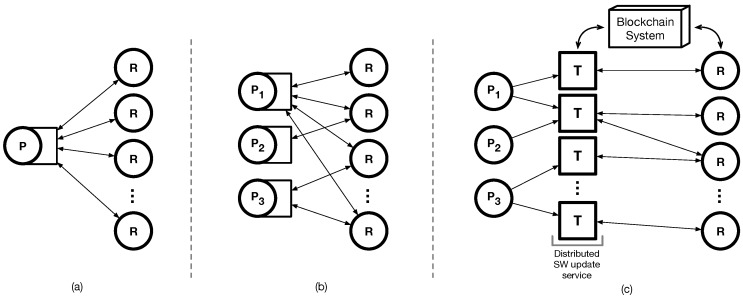
Models of software update: (**a**) the one-to-many model; (**b**) the many-to-many model (multiple providers or vendors); (**c**) the proposed distributed software update model, where *P* is the provider or manufactorer, *R* is the recipient device, and *T* is the patch transporters.

We propose PatchTransporter, an incentivized patch delivery approach based on the blockchain system. The model for software updates can be categorized as in  Figure 1. First, a single provider is operated for software update management and it is in charge of updating all the devices (Figure 1a). When a device has multiple software components, multiple providers handle the software updates to multiple devices at the same time (Figure 1b). In both cases, a provider needs to carefully manage its own management server for software updates with high availability and scalability, which is challenging for small manufacturers. Thus, our approach is to provide a distributed service of software update for the WSN/IoT environments (Figure 1c). We first decouple patch generation and delivery. As a distributed software update service, the self-interested worker, called ‘transporter’, fetches the patch and securely delivers it to devices in a distributed way. We also use the blockchain system to supervise the participants to follow the rules for delivering patches at the core. A participant can achieve its goal, i.e, a patch or a fee, only when it behaves properly. With a solid reward system, the transporters competitively find targets and deliver the patch, thus the efficiency of patch delivery can be maximized.

**Figure 2:**
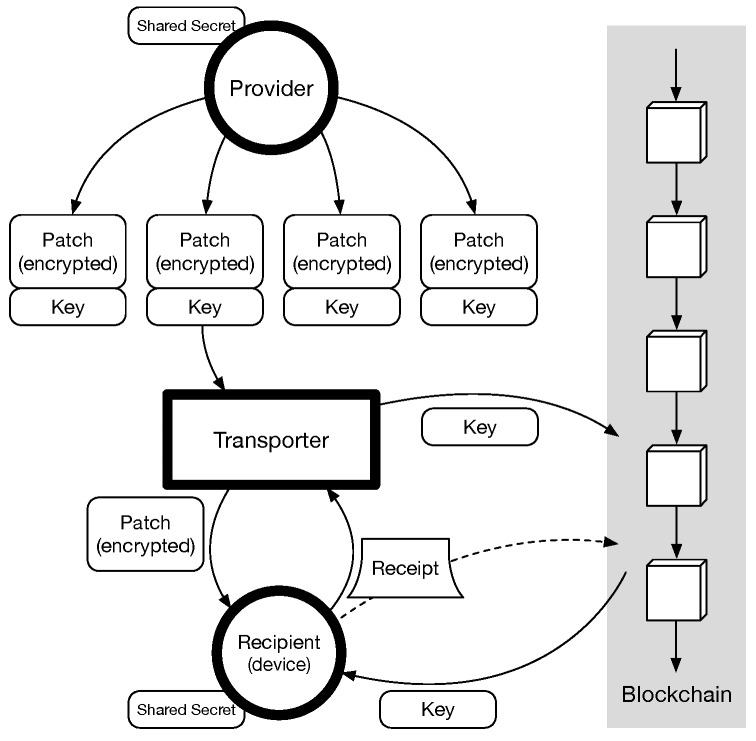
PatchTransporter process.

We define the roles of participants to describe our approach. A *provider* prepares a software patch as a patch package, which is a deliverable unit of a patch and doubly encrypted. A *transporter* identifies the targets of a patch packages and then delivers to a target *recipient*. If the patch package is properly delivered, the recipient pays a fee to the transporter.  Figure 2 shows the overall process of PatchTransporter.

We define the following properties to ensure the delivery system is correctly operating.

**Definition** **1** (Faithful Delivery)**.** If a transporter picks up a patch package from a provider and a target of the package is identified, the transporter should deliver the package without any modification.

**Definition** **2** (Authenticated Origin)**.** A recipient should be able to authenticate the origin of the delivered patch package.

In order to satisfy the properties, we encrypt the patch package with the key *s*, which is a preshared key only between the provider and the recipient. If a device consists of software components from multiple vendors, it may also have a set of *s*. The integrity of a patch package, including a software patch and metadata, is also protected by a keyed hash with *s*. Finally, we also define the property for the fairness of the patch delivering system.

**Definition** **3** (Fair Reward)**.** A transporter should be paid by a predefined fee for every faithful delivery of a unique patch package, whose origin is authenticated.

We employ blockchain to guarantee Fair Reward. For this purpose, the patch payload in the *i*-th patch package is doubly encrypted by ${\mathrm{key}}_{i}^{E}$ as well as *s*. The transporter hides its decryption key ${\mathrm{key}}_{i}^{D}$ from the recipient when delivering the patch package. The recipient checks the integrity of the delivered patch package and authenticates the origin with *s*. If it is valid, it issues a *receipt* embedding a smart contract code and a fee. The smart contract code of the receipt is initialized with the hashed value of ${\mathrm{key}}_{i}^{D}$, and it is set to he transfer the fee to who reveals ${\mathrm{key}}_{i}^{D}$. Hence, the transporter can get the fee by sending a transaction with publicly revealing $k_{i}$, so that the recipient can learn it at the same time. That is, the receipt deployed in blockchain enables the atomic exchange of $k_{i}$ and a fee between the transporter and the recipient.

*Attack model*. In this paper, we focus more on the domain-specific attacks that can be leveraged in the real implementation with the blockchain system than the attacks of communication itself, e.g., jamming, since the protection against the generic network attacks has already been explored in a number of research papers. In our attack model, we assume that any participant can perform malicious behaviors to fulfill their goals at the minimized cost. If the transporter is malicious, it can forge the delivery package in order to get the fee without delivering a legitimate package or in order to consume the computing resource of the recipient. It can also perform relay attacks by sending the same package repeatedly. If the provider and the recipient are malicious, their goals are to let the transporter deliver the package without paying the fee. Thus, we assume that the malicious provider and recipient can exchange a small message hidden from the transporter. Additionally, an outside attacker can observe the process in the wireless channel and the blockchain to intercept the fee between the recipient and the transporter. Based on this attack model, we analyzed the proposed process with the vulnerabilities specified in the literature of escrow protocol [23], blockchain [24,25,26], and smart contract [27,28,48], in order to identify the possible attacks.

- *Malformed Receipt*. In activating the receipt on the blockchain, two steps are required. First, the transporter or the recipient deploys the receipt on the blockchain system. Second, the transporter claims the fee by revealing the decryption key, ${\mathrm{key}}^{D}$. However, the recipient creates the smart contract code in the receipt, and the transporter cannot modify it since it is encapsulated as a signed transaction. Thus, if a recipient is malicious, it can deceive the transporter by providing a malformed smart contract code in the receipt.
  − *Revealing key with exception*. The transporter performs the second step by publishing a redeem transaction that reveals the decryption key to claim the fee. If a malicious recipient injects an illegal instruction to the smart contract code of the receipt, the redeem transaction of the transporter is finished as a failure (and revert all the changes). However, the transaction with the key is already transmitted [27]. The malicious recipient can learn the key from the transaction without paying any fee.  
  − *Gas-consuming receipts*. This is an Ethereum-specific problem. When executing a smart contract in Ethereum, the caller should pay the cost of execution, called ‘gas.’ More specifically, each low-level instruction has its own gas cost depending on the impact on computing power/storage of blockchain (For example, the gas cost of ADD is 3 but that of CREATE, which creates a new account, is 32,000 according to [41].). If PatchTransporter is implemented on the Ethereum system, the transporter pays the gas costs to execute the smart contract code in the receipt. However, a malicious recipient can inject the gas-costly operations in the receipt and make the transporter pay the high costs in redeeming the key. (Recent research [28] shows seven gas-costly patterns in the smart contract of Ethereum.) Furthermore, if the transporter could not properly expect the cost of the gas-costly operations, the execution of the redeem transaction is aborted with revealing the key due to the insufficient gas exception or the excess of the block gas limit.
- *Fee Interception*. Unless the receipt strictly checks the beneficiary, the result of the receipt becomes transaction-ordering dependent [24]. In blockchain, the miner who creates the block can select the order of transactions. Thus, if two conflicting transactions occur at the same time, the winner is dependent on the miner’s decision. In our case, as soon as the transporter sends the transaction to redeem the key, an attacker can send a new transaction with the same key learned from the transporter’s transaction. If the miner selects attacker’s transaction first, the attacker can intercept the fee.
- *Transporter Outcast*. Another reason for the double encryption of the package is to make every package unique. Thus, if the transporter does not participate in the generation of patch package, then the provider uses its own key pool (the Key Pool method will be explained in Section 4.1). In the case that the provider and the recipient are collaboratively compromised, the provider can secretly hand over the other decryption key, ${\mathrm{key}}_{i}^{D}$, to the recipient. Then, the transporter delivers the patch package but cannot get the receipt for the delivery, since the recipient already knows all the keys for decrypting the patch, *s* and ${\mathrm{key}}_{i}^{D}$.
- *Denial of Service Attack on Recipients*. Since the recipient is an energy-constrained node, a malicious transporter may send a forged package to consume the energy of recipient in processing the forged package. In particular, when the Key Registration method is used, which will be explained in Section 4.1., the transporter generates a pair of public key and private key and registers a key for encryption and a hash value of the other key for proving the decryption key. Since the provider requests only a key in the plain text form, a malicious transporter may register a key and a hash value of a wrong key. In this case, the malicious transporter may illegally take the fee while the recipient consumes the energy to perform public key decryption with a wrong key.

We design PatchTransporter to satisfy all the properties under the consideration of these security attacks. Thus, we devise the structure of patch package and the receipt, and the receipt assessment and key registration for the transporter. The detailed process is explained in Section 4 and the specification of the whole system is given in Section 5.

*Example scenario*. For an example scenario of PatchTransporter, let us suppose that a critical security vulnerability is found on a version of the common bluetooth library, and a number of the old IoT smart locks are using the library to communicate with the smart keys of the customers. Thus, an unauthorized attacker may open the smart lock by using the new vulnerability. However, the manufacturer of the smart lock does not have a full IP list of every device and reliable network service infrastructure to support the urgent security patch. Then, our approach is advantageous. First, the provider (the manufacturer) generates patch packages and encrypts each package using the keys registered by transporters, and then posts the packages on a patch hub website. Then, the transporter (as an automated agent) fetches the packages and quickly scans the compatible IoT smart locks in the Internet by using pre-searched results (e.g., Shodan [49]), or rapid online search (e.g., ZMap [50]), or its own management list. If the transporter finds a matched smart lock device, it transmits the package to the recipient device. The recipient smart lock verifies the package. If it is legitimate, the smart lock creates a receipt to the transporter by using its deposit for the delivery fee. The transporter then also checks the validity of the receipt. If the receipt is valid, the transporter submits a pair of receipt and the decryption key revealing transaction, which is generated by the transporter. At the same time, the transporter can hand over the decryption key for the package, or the recipient device can learn it from the revealed key in the blockchain. In this process, the customer of the smart lock is willing to pay the fee for rapid update since the vulnerability is critical to home security (Please note that financing of the fee management, e.g. the co-payment of the fee with customer and manufacturer, or the dynamic fee adjustment, is not handled in this paper.).

## 4. Design

**Figure 3:**
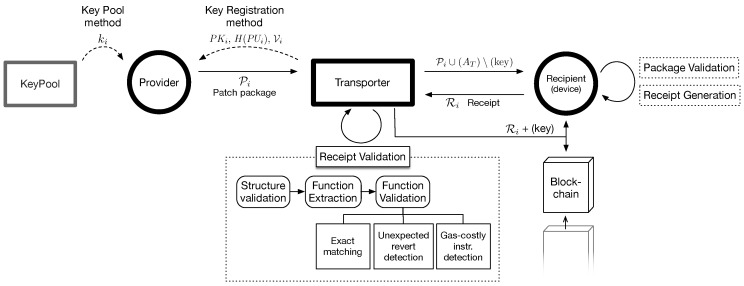
The detailed process of delivering a patch.

In this section, we describe the detailed process of PatchTransporter. The whole process can be explained in the following steps: (1) patch package preparation (Section 4.1); (2) package delivery and validation (Section 4.2); (3) receipt generation (Section 4.3); (4) receipt validation (Section 4.4) and (5) redemption and key disclosure (Section 4.5).  Figure 3 shows the detailed process that will cover in these steps.

### 4.1. Patch Package Preparation

A provider prepares patch packages when the provider needs to deliver a patch, denoted by *u*, to the recipients. We assume that a provider of a software component shares a secret key *s* with the recipient devices that installed the components (To securely manage and share the key, the conventional key management methods [51] can be applied.). First, the provider encrypts the patch *u* with *s*, E(s,u). To achieve the Fair Reward property, each patch package needs to be unique. Thus, we doubly encrypt *u* in a patch package (1) to authenticate the provider and the patch itself, (2) to create a unique package from the same *u*, and, optionally, (3) to give the control of patch package to the transporter (in order to prevent the Transporter Outcast attack of Section 3). The second encryption on E(s,u) has two options. If we can ensure the provider is benign, we can use a simpler method based on a key pool, the Key Pool method. Otherwise, we need to use the Key Registration method, where the transporters generate keys and register them to the provider. Note that this is a trade-off decision. The latter method is secure against Transporter Outcast, but two performance issues arise: (1) the recipient, which is a WSN or IoT device, has to perform more resource-expensive operations of public key cryptography; and (2) the provider cannot pre-generate patch packages.

*Key Pool Method*. (${\mathrm{key}}_{i}^{E}={\mathrm{key}}_{i}^{D}=k_{i}$) The provider first generates a key pool. The provider then composes a unique patch package by encrypting a patch and its metadata with a distinct key from the key pool. More formally, let $k_{i}\in K$ be a generated key in a key pool K. The encrypted payload is encrypted again with $k_{i}$, $E\left(k_{i},E\left(s,u\right)\right)$. A patch package $P_{i}$, is composed of a 4-tuple:

(1) $$P_{i}=\left(k_{i},\;H\left(k_{i}\right),\;E\left(k_{i},E\left(s,u\right)\right),M_{i}\right),$$

where $H(k_{i})$ is a hashed value of $k_{i}$ and $M_{i}$ is the metadata for $P_{i}$. The metadata M consists of a nonce, N, the specification of target, S, and a timestamp for the creation time of *u*, $T_{u}$. In order to check the integrity and the freshness of the payload, M also has the keyed hash on the encrypted payload, the nonce N, and the timestamp with *s*, $H(s\|S\|N\|T_{u}\|H\left(k_{i}\right)\|E\left(k_{i},E\left(s,u\right)\right))$, where ‖ is a concatenation operator. To sum up,

(2) $$M_{i}=(N,S,T_{u}{,\;H(s\|S\|N\|T}_{u}\|H\left(k_{i}\right)\|E\left(k_{i},E\left(s,u\right)\right)).$$

*Key Registration Method*. (${\mathrm{key}}_{i}^{E}=PK_{i},{\mathrm{key}}_{i}^{D}=PU_{i}$) In the key registration method, the provider does not generate a key pool. Instead, a transporter generates a public/private key pair for *i*-th patch package, i.e, $PU_{i}$ and $PK_{i}$, respectively, and registers a key for encryption and a hashed key for decryption, which will be revealed at the end of the process. Even though the transporter presents only a key in plain text, the provider and the recipient should be able to ensure that the transporter will reveal the correct key pair. Thus, the provider requires the transporter to generate a signature for a challenge. If the signature can be verified with the revealed decryption key at the key validation step, the recipient can ensure whether the revealed key is the right pair of the encryption key. However, in Ethereum, the signature for a message is generated by using both a public key and a private key, and it is validated (via calling ecrecover) by returning its public key (in the Ethereum address form). Thus, in order to employ the Ethereum-specific signature, we choose the private key for encryption and the public key for validation and decryption. To do so, the provider gives a challenge H(N‖s) to a transporter, and then the transporter registers $PK_{i}$, $H(PU_{i})$, and the signature for the challenge, $V_{i}$. The provider uses $V_{i}$ to check the revealed key is a proper pair of $PK_{i}$ in the receipt of Section 4.3. That is, it uses $PK_{i}$ and $H(PU_{i})$ instead of $k_{i}$ and $H(k_{i})$, respectively, for creating the package. Thus,

(3) $$P_{i}=\left(PK_{i},\;H\left(PU_{i}\right),\;E\left(PK_{i},E\left(s,u\right)\right),\;M_{i}\right),$$

and its metadata includes the signature $V_{i}$,

(4) $$M_{i}=(N,\;S,\;T_{u},\;V_{i}{,\;H(s\|S\|T}_{u}\|H\left(PU_{i}\right){\|V}_{i}\|E\left(PK_{i},E\left(s,u\right)\right)).$$

The crux of this method is that the transporter has control of decrypting the patch since it keeps $PU_{i}$ as a secret while the secure binding between the encryption and decryption keys is guaranteed.

### 4.2. Package Delivery and Validation

After the provider prepares the patch packages, transporters pick up the packages for delivery. According to the target specification in $M_{i}$, a transporter finds the target devices of patch packages. The transporter can carry out the delivery of packet packages in their own ways. Obviously, the simplest way to identify a number of the target devices is the wide-range Internet scanning, e.g., Shodan and ZMap.

Once the transporter finds a target device (a recipient), it delivers a patch package without the key but with the blockchain address of the transporter. That is, $P_{i}\cup \left(A_{T}\right)\backslash \left(k_{i}\right)$ or $P_{i}\cup \left(A_{T}\right)\backslash \left(PK_{i}\right)$, where $A_{T}$ is the address of transporter, for the key pool method or the key registration method, and ∪ and \ is union and minus operators for a tuple, respectively, which are used to add and remove an element from the duple. Then, the recipient verifies $M_{i}$. First, it matches S with its own specification and then it checks the integrity of the patch package by comparing the hash value stored in $M_{i}$ with the hash value calculated from $T_{u}$, the encrypted payload, and its own preshared secret, *s*. Since *s* is preshared secret with the provider, if the package is modified during delivery or is forged by a malicious provider, two results cannot be matched. Finally, it checks the patch is delivered within a golden time, which is the time duration of the patch being effective, by $(T_{\mathrm{now}}-T_{u})<G$, where $T_{\mathrm{now}}$ is a timestamp of current time and G is the golden time (delivery time out) of a patch. If every condition is satisfied, the receiver device prepares a receipt, $R_{i}$, for $P_{i}$.

### 4.3. Receipt Generation

If the recipient could successfully validate the received package, the last operation is the exchange of the decryption key and the fee between the transporter and the recipient. During this process, the Fair Reward property should be satisfied under the consideration of the possible attacks in Section 3. Thus, we devise a receipt as the smart contract code of the following requirements:

**R1:**  It should store the hash value of the decryption key, i.e., $H(k_{i})$ or $H(PK_{i})$, to check whether the revealed decryption key is correct.
**R2:**  It should clearly indicate and check the beneficiary of the fee.
**R3:**  It should be optimized to minimize additional cost to execute the code.
**R4:**  It has to provide a standard interface for registering the hash value of the key and the beneficiary, and redeeming the key.

Representative bitcoin systems, e.g., Bitcoin and Ethereum, support smart contracts but with different functionality. The receipt can be implemented on both the Bitcoin and Ethereum system, but we first assume the Ethereum smart contract for the receipt. Algorithm 1 shows the pseudo code for the receipt. The receipt smart contract has a constructor, PatchReceipt and a redeem function proofOfKey. When the receipt is deployed, it is constructed with the hashed key value, $H(k_{i})$ or $H(PU_{i})$, the transporter’s address as the beneficiary and, optionally, $V_{i}$ and the challenge H(N‖s). In the next step, the redeem function proofOfKey is called by the transporter with the decryption key, which is publicly revealed. It then checks that the hash value of the decryption key is the same as the stored value. In addition, for the Key Registration method, it also checks whether the generated key from $V_{i}$ is the same as the given key (via verifySign). In other words, it confirms that the decryption key provided by the transporter can decrypt the package. The verifySign function in Ethereum can be implemented by using ecrecover, which is a precompiled smart contract at the address 0x01 to validate a signature by recovering a public key from a given signature. Additionally, in order to prevent the Fee Interception attack, the beneficiary of the fee is registered at the receipt constructor and is checked before transferring the fee.


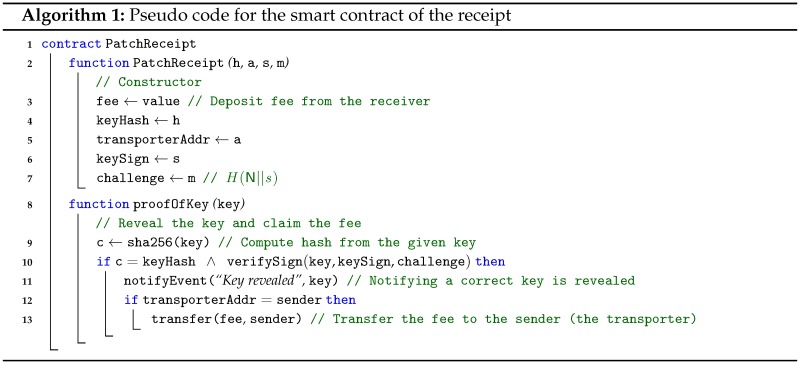


For the Bitcoin blockchain, we can use a Bitcoin Script [52] to implement a smart contract. A transaction, $tx_{I}$, in Bitcoin can use the fund at the output of another transaction, $tx_{O}$, if the concatenation of the output script of $tx_{O}$ and the input script of $tx_{I}$ is evaluated as true. Thus, the recipient can make the receipt as an transaction containing the fee, and the receipt transaction has the condition of revealing the correct key in the script. The output script for the receipt transaction contains the hash value of the decryption key, i.e., $H(k_{i})$ or $H(PU_{i})$, and checks whether the corresponding input script of the following transaction has the decryption key. For example, the output script for the receipt transaction has the additional opcodes: “$\mathrm{OP}\_\mathrm{SHA}256\;\;\mathrm{OP}\_\mathrm{PUSHDATA}1\;\;0x20\;\;H(k_{i})\;\;\mathrm{OP}\_\mathrm{EQUAL}$”, where $H(k_{i})$ can be replaced with $H(PK_{i})$ if we use the Key Registration method. The corresponding input script starts with “$\mathrm{OP}\_\mathrm{PUSHDATA}1\;\;|k_{i}|\;\;k_{i}$”, where $|k_{i}|$ is the length of $k_{i}$, and $k_{i}$ can be also replaced with $PU_{i}$ for Key Registration method. The whole execution of the concatenation of output and input script results in true only if the decryption key is correct, and then the transporter can transfer the fee to herself (Note that since the signature validation process of $V_{i}$ in the Key Registration method is not compatible between Ethereum and Bitcoin, we focus on the Ethereum approach in this section).

The recipient can directly submit the receipt to the blockchain system or the transporter can relay the receipt to the blockchain system as the form of a raw transaction. Since every valid transaction has been signed by the initiator, the integrity of the transaction is kept in any form.

### 4.4. Receipt Validation

After the recipient generates the receipt, the transporter sends the redemption transaction that reveals the decryption key. However, due to the Malformed Receipt attack described in Section 3, the transporter should validate the smart contract code in the receipt transaction. If the transporter relays the receipt to blockchain, it can validate the receipt before deploying it. Even if the receipt is already deployed, the transporter can validate the smart contract code of the receipt, since it is publicly downloadable. However, the smart contract code is complied to a binary form, e.g., EVM bytecode in transactions, the transporter needs to validate the binary code of smart contract. Note that, since the Malformed Receipt attack mostly focuses on the Ethereum system, we describe the validation of Ethereum smart contract code in this section.

The complete semantic validation of the low-level EVM bytecode requires a symbolic execution system [24] of heavy load, but it is yet overkill for transporters. Thus, we use a syntactic approach to prevent the attacks related to the receipt. Since the disassembly of EVM bytecode is straightforward, we assume the transporter validates the smart contract code of the receipt at the EVM assembly level in the following steps. Algorithm 2 also shows the pseudo code for the receipt validation, the entry point of which is ReceiptValidate.


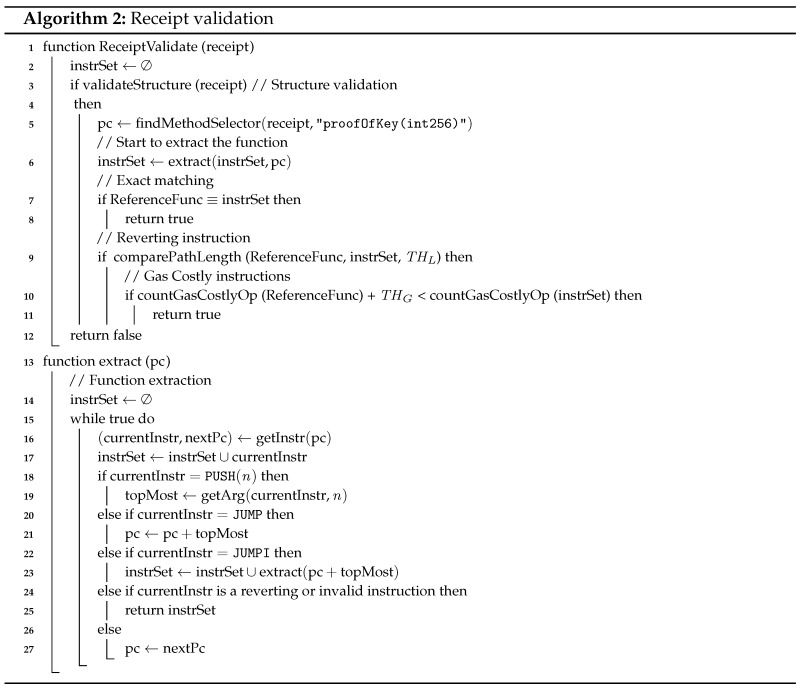

1. *Structure validation*. First, the transporter checks the smart contract code has valid interfaces. The bytecode generated from Solidity, which is a representative programming language for Ethereum, has two parts: one for contract deployment (and constructor) and the other for remaining functions. The latter part starts with a method selector, which compares the calldata with its function signatures to select a function to execute (jump to the address of the function code). The function signature is the 4-byte truncated values of SHA3 of function descriptions. For example, the function description of proofOfKey is “proofOfKey(uint256)” since it takes a single argument of uint256 for the decryption key, and, then, the function signature is calculated by sha3(proofOfKey(uint256))${|}_{4}$, where ${|}_{n}$ denotes the truncation of *n* bytes, i.e., “0x9086f6fc.” Therefore, the transporter can check the receipt has the correct interface by identifying the function signatures, e.g., 0x9086f6fc, in the method selector.
2. *Function extraction*. The transporter then extracts the code of the redemption function, proofOfKey. From the jump address of the method selector, it recursively follows the control flow until the epilogue of the function or revert instruction. The function extract in Algorithm 2 shows the extraction process. The getInstr function gives the current instruction currentInstr at the address of pc and the address of the next instruction, nextPc. To calculate the address of jump destination, we keep the value at the top of the stack in topMost (Line 18). This approach needs the trace forking at a conditional branch JUMPI to follow both true and false branch (Line 22).
3. *Function validation*. On the extracted redemption function, the transporter can apply the following validation.
  - *Exact matching*. In most cases, the smart contract part of the receipt is the same. The variant part of the receipt is the arguments of the constructor, such as the hash of decryption key and the transporter address. Thus, the transporter can use the invariant code part as a reference code (ReferenceFunc in Line 7). If the two codes are the same, the code is trustworthy and no more validation is required. However, depending on the compiler version and optimization options, different bytecodes can be generated from the same source code. Thus, the transporter continues to check the following problems in the receipt.  
  - *Unexpected revert detection*. To prevent the attack of revealing key with exception in Section 3, the transporter checks there is unexpected illegal opcodes in the redemption function, since an illegal opcode can be used to revert the execution. For the purpose, we compare the length of paths in the reference function ReferenceFunc and the target code (Line 9). We identify every path in each code and find a matched path of the same length with the margin of $TH_{L}$ (in comparePathLength). Since a reverting or invalid instruction stops the extraction of the path, it causes the difference of the extracted path. If a path with a significantly different length is found, the transporter rejects the receipt.  
  - *Gas-costly instruction detection*. Likewise, the gas-costly instructions can incur additional costs when executing the function or may lead to unexpected stop of execution due to insufficient gas. Thus, the transporter counts the number of gas-costly instructions in the function, such as SLOAD, SSTORE, BALANCE, and SUICIDE [26,28] and compare it with that of the reference function (Line 10). If the difference is higher than threshold $TH_{G}$, the transporter also rejects using it.

### 4.5. Redemption and Key Disclosure

If the transporter can validate the smart contract code of the receipt, it then sends the redemption transaction with revealing the decryption key $k_{i}$ or $PU_{i}$. For example, the transporter calls proofOfKey with the decryption key in the Ethereum. If the decryption key and the beneficiary are correct, then the receipt smart contract code sends the fee to the transporter. Through this process, the recipient can learn the decryption key and finally gets the patch *u* from the package by $D(k_{i},D\left(s,u\right))$ or $D(PU_{i},D\left(s,u\right)).$

## 5. Formal Specification

We developed a formal specification of PatchTransporter in the TLA^+^ specification language [53]. Through the specification, we can precisely define the behavior of PatchTransporter and perform model checking to verify the correctness. In this section, we present a formal specification on steps of PatchTransporter with explanation, and then we describe the results from the model checking on the specification and the simulation with the implementations in Section 6. The specification of this section in this section is shown as excerpts from the complete specification, which can be found in Appendix A.

### 5.1. Overview

**Figure 4:**
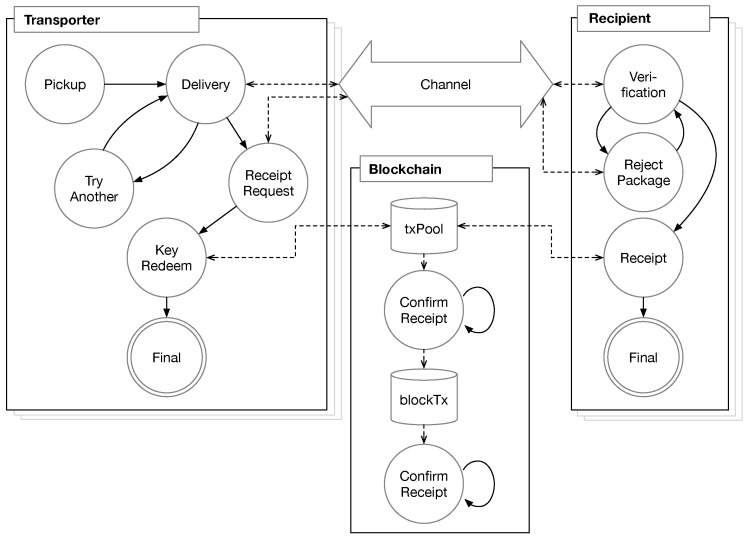
Abstracted relationship of the participants of PatchTransporter in the specification (the provider is omitted). The solid line shows the state transition and the dotted line shows the movement of data.

In PatchTransporter, we have the four kinds of participants: the provider, the transporter, the recipient and the blockchain system. For the delivery process, the provider is an offline entity and the rest are online entities.  Figure 4 shows the relationship among the online participants. In the specification, each entity has its own steps as shown in  Figure 4. A provider has a step for generating patch packages. The transporter and the recipient also has its own steps for delivering and processing patch packages, and buffering storage to store the package. The transporter communicates with the recipient though a *channel*. The blockchain is an independent entity and has storage of two kinds: one for unconfirmed transaction or smart contracts, *txPool*, and the other for confirmed ones, *blockTx*.

In the specification, we describe the behavior of each participant in steps. Each step is represented as a TLA^+^ action formula. The action shows the change of individual state (i.e., variable) before and after the action. For the purpose, the action formula has primed and unprimed variables. That is, the primed variable shows the new value after the action and the unprimed variable represents the current value. Hence, each step (or action) may have unchanged variables, i.e., $v^{\prime }=v$. The keyword unchanged indicates the unchanged variables in the action formula.

The specification starts by describing an initial step. Then, it describes the transitions among the actions from the initial state. In this specification, we describe all the possible transitions at the *Next* action formula in Section 5.2 with OR-relations (disjunction), thus the formula of each step generally has two parts: (1) the enabling conditions, which should be satisfied to enter the step and (2) the variable changes. More detailed information on TLA^+^ can be found in [53,54].

In this way, the specification shows every possible change of the system. Thus, it can benefits from removing ambiguity, but it is also advantageous in the model checking process of Section 6. The model checker traverses every possible combination of transitions and changes and finds out deadlock cases (no possible transition), and confirms that the given properties are satisfied. We will first describe the initial state and the possible transitions in the following section and then explain each step of the participants.

### 5.2. Transitions

In the initial step, *Init*, we set a default value for each variable. The formulas *InitProvider*, *InitTransporter*, *InitRecipient*, and *InitBlockchain* are of initial conditions for providers, transporters, recipients, and the blockchain system, respectively. *channel* is a variable to contain communication message between transporters and recipients. *Init* is shown as follows:


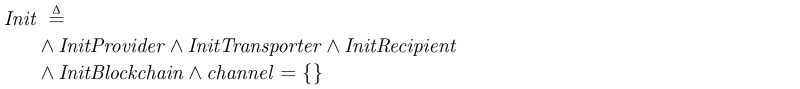


We then describe the state changes. The standard form for overall state changes in TLA^+^ is $Init\wedge □{\left[Next\right]}_{vars}$. All of the possible changes of states from *Init* are described in *Next* in a disjunction as follows:


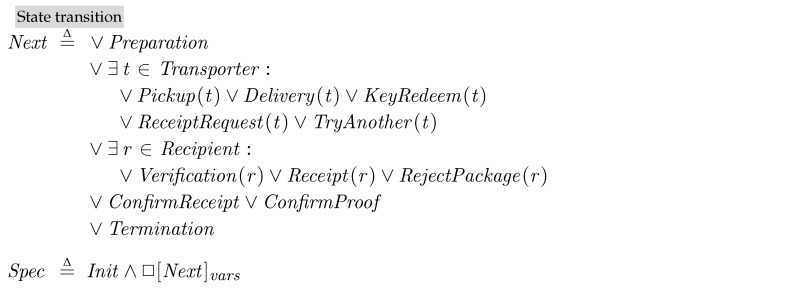


As shown in  Figure 4, we assume that the specification describes a process for applying a single kind of patch packages when there are multiple transporters and recipients. From the *Preparation* step, where the provider prepares patch packages, depending on the current states, a certain transporter (∈Transporter) or a certain recipient (∈Recipient) carries out the operations. We assume that the blockchain system independently performs its duty, which is confirmation of incoming transactions. Whenever there are complete transactions (a pair of input and output) in the pool of unconfirmed transactions, *txPool*, the blockchain system checks their validity and confirms them in *ConfirmReceipt* and *ConfirmProof*. The final step *Termination* describes the termination condition of PatchTransporter, such as all the recipients successfully get patches.

### 5.3. Providers

The provider generates patch packages. *Preparation* is a conjunction of TLA^+^ actions. Since the purpose of the specification is to describe the behavior of the system, describing all the internals of cryptographic operations is overkill. Thus, we represent the cryptographical operations, such as key generation, encryption, and hash operations, as external oracles in the specification. We provide the values as tuples, sets or mapping tables (or record type) from outside of specifications. The specific values for the external oracles are given in model checking to test the system with various configurations. Here, we assume that the Key Registration method is used in preparing packages. Thus,  transporters register their pairs of private keys, the hash value of public keys, and the signature on the given challenged using the both keys (Section 4.1) in *regKey*. As one of the external oracles, the mapping table *EPAYLOAD* is used to find an encrypted patch payload from a private key. *NODE_TYPE* is a set of target recipient IDs of this package. *checksum* and *checksumId* are also used in a later phase for a recipient to verify that the received package is valid. Finally, the generated patch package is publicly posted to be picked up by transporters.


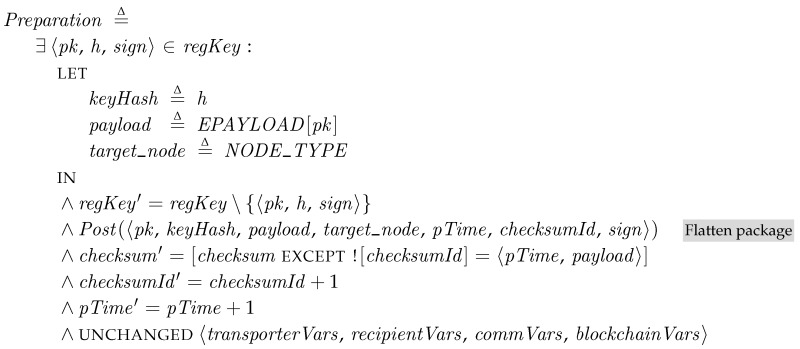


### 5.4. Transporters

A transporter has internal states that are used to distinguish the current step of each transporter in the enabling conditions. Initially, a transporter is at *T_Waiting*. The transporter then progresses through *T_Delivering*, *T_VerificationWaiting*, *T_ReceiptWaiting*, *T_MiningWaiting*, and *T_Final*. The actions of transporters are defined depending on the internal states.

*Pickup*. First, a transporter in *T_Waiting* gets to the *Pickup* step, in which it picks up a patch package from *patchPool* and stores it to *tBuffer*. The state then is changed to *T_Delivering*. The code for the specification as follows:


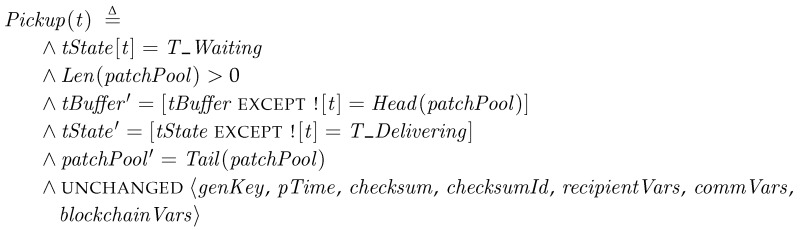


As we explained in Section 5.1, the first two lines are the enabling conditions, tState[t]T_Waiting and Len(patchPool)>0, and the following three lines show the change of variables to primed ones. The unchanged variables are indicated in the unchanged operator.

*Delivery*. When a transporter in the *T_Delivering* state, it discovers a target recipient and delivers the patch package to the recipient. We define a helper operator *Send*. It sends a message of record type. We denote the field of a message for source, destination, type, and payload by *src*, *dst*, *type*, and *data*, respectively. The transporter transmits the stored package except the key, which will be revealed in a later *KeyRedeem* step.


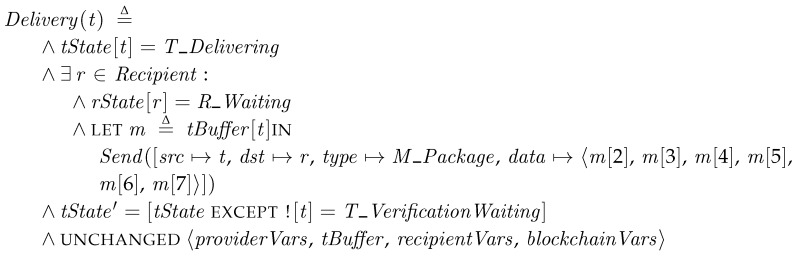


After sending the message of the patch package, the transporter enters to the *T_VerificationWaiting* state. It waits for the reply from the recipient.

*ReceiptRequest*. If the transporter receives the reply from the recipient and the result is positive (*M_OK*), then the actions of *ReceiptRequest* are performed. The recipient message means that the delivered package is valid and it is ready to create the receipt (in a raw transaction or smart contract form). Thus, the transporter requests the receipt by sending a *M_ReceiptRequest* messages. We found that this step is required through model checking in order to avoid deadlocks with multiple transporters and recipients. Additionally, in the following specification, we use *RecvInadditionSend* operation to removing the received message and send a new message.


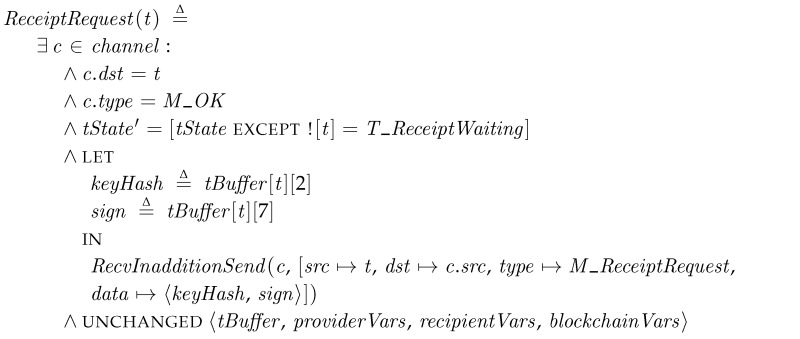


*TryAnother*. The recipient may determine that the received package is not valid. Then, it rejects the package by sending a *M_NO* message to the transporter. *TryAnother* is a phase for handling the reject message. When the transporter receives the *M_NO* message at *T_VerificationWaiting*, it goes back to *Delivery* and finds another candidates.


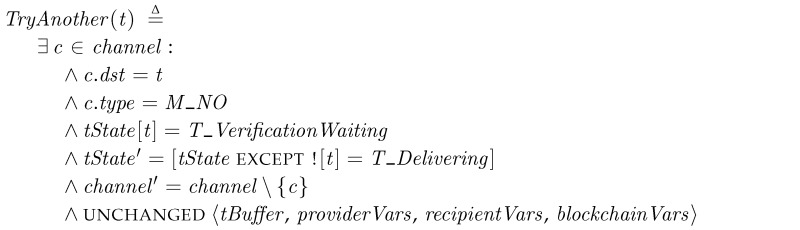


*KeyRedeem*. Once the recipient publishes the receipt to the blockchain (with or without relaying by the transporter), the transporter can find the receipt transaction or smart contract is confirmed by the blockchain system (*blockTx*) (at *ConfirmReceipt*) and then enters to *KeyRedeem*. The receipt validation in Section 4.4 is performed by *ValidateReceipt*. Since the receipt does not contain the real opcodes of smart contract in this specification, we simplify the validation as checking the beneficiary is correct. If the receipt is valid, the transporter sends the redemption transaction to reveal the public key. The operation, *Proof*, is used for the purpose.


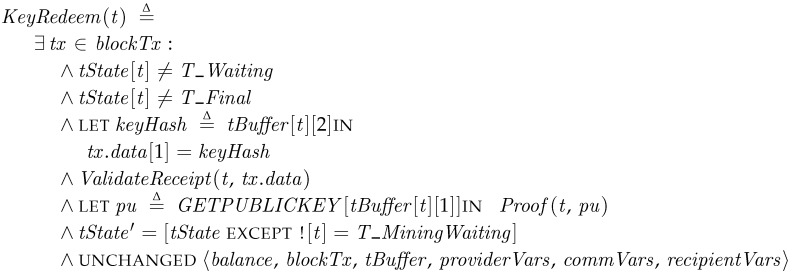


The transporter in *T_MiningWaiting* then waits for a miner (or a verifier) in the blockchain system to confirm the transaction and records it to blocks. Once a miner confirms the receipt and this proof transaction at *ConfirmProof*, which will be explained in later, the transporter gets the fee for successful delivery and it reaches the final state, *T_Final*.

### 5.5. Recipients

A recipient also has internal states: *R_Waiting*, *R_Verifying*, *R_RequestWaiting*, *R_Paying*, *R_Final*. It starts at *R_Waiting* and waits for a transporter.

*Verification*. This step of a recipient is triggered by a transporter. When a transporter finds the recipient and sends a package to it, the recipient tries to check whether the package is valid. The operator *VerifyMeta* checks the parameters of the received package in *c.data*. The verification of Section 4.2 requires cryptographic operations. However, as we discussed in Section 5.3, we assume that external oracles, which are given as mapping tables, can perform the cryptographic operation instead. Thus, *VerifyMeta* checks the received parameters and the return value of the external oracles are equal. In addition, the recipient checks its ID is in the set of target nodes of the package, *c.data*[3]. If all the parameters are valid, the recipient stores the received message and sends a *M_OK* message to the transporter, and then waits for receipt request in the *R_RequestWaiting* state. Otherwise, it rejects the package by sending a *M_NO* and remains at the same state.


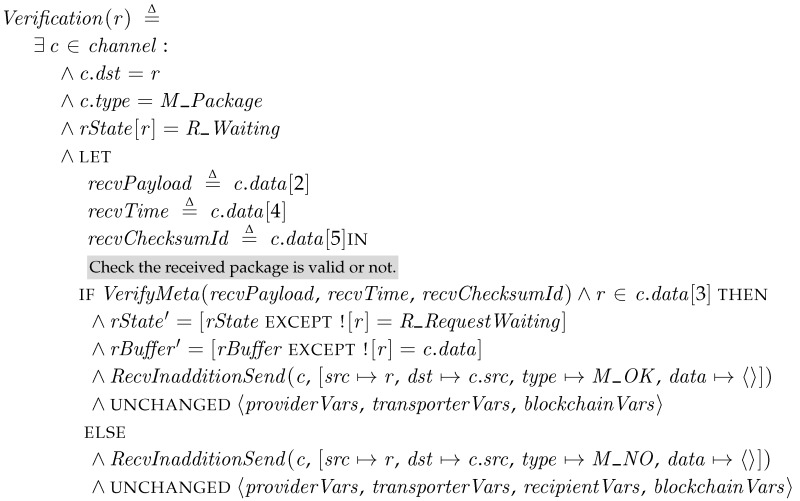


*Receipt*. When the transporter requests the receipt for the package by sending a *M_ReceiptRequest* message, the recipient creates a receipt and enters to the *R_Paying* state, at which the recipient waits that the transporter reveals the key and the blockchain system confirms it. The operator *Pledge* generates a receipt with the hash value of the key, the signature, and its beneficiary of the fee. In this model, we assume that *Pledge* directly publishes a receipt transaction to the blockchain, but it can be also implemented as the transporter relays the receipt transaction to the blockchain.


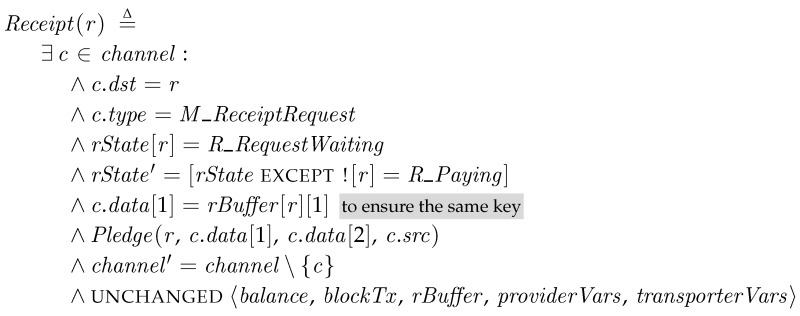


*RejectPackage*. When a transporter sends a package and the recipient already has a received package, the recipient notifies the transporter to reject the package by sending the *M_NO* message.


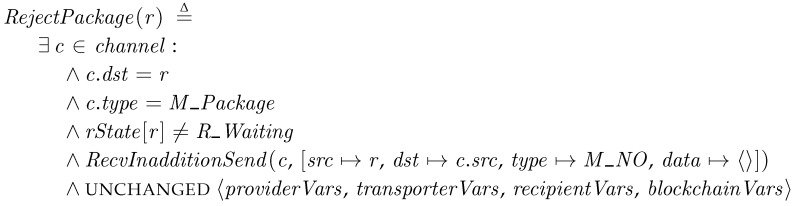


### 5.6. Blockchain and Termination

*Blockchain Confirmation*. A whole blockchain system is complicated, thus we model only its essential part for our system. In our specification, the blockchain system is represented in independent steps of *ConfirmReceipt* and *ConfirmProof*. Whenever there is a valid transaction, the blockchain system confirms and moves it to *blockTx*, which is a set of confirmed transactions in the blocks. In checking a receipt transaction, the blockchain system checks the recipient has enough balance to pay the fee. If the recipient can pay the fee, then it moves to *blockTx*. In *ConfirmProof*, the blockchain system finds a pair of transactions that satisfy the following two conditions: (1) the pair of transactions are of complementary types of *TX_In* (from *blockTx*) and *TX_Out* (from *txPool*); (2) the hash value of the data in the *TX_Out* transaction is equal to the data in the *TX_In* transaction; and (3) the signature in the *TX_Out* transaction is validated with the key in the *TX_In* transaction. In other words, the correct key should be revealed in the *TX_In* transaction. For this purpose, we use external oracles *KEYHASH* and *SIGNVERIFY*. Then, the fee is transferred to the beneficiary indicated in the receipt transaction. Since we assume that the blockchain system is available at all times, the participants can learn the updated information eventually. Thus, for simplicity, the states of the corresponding transporter and recipient are changed to the final state, i.e., *T_Final* and *R_Final*, respectively.


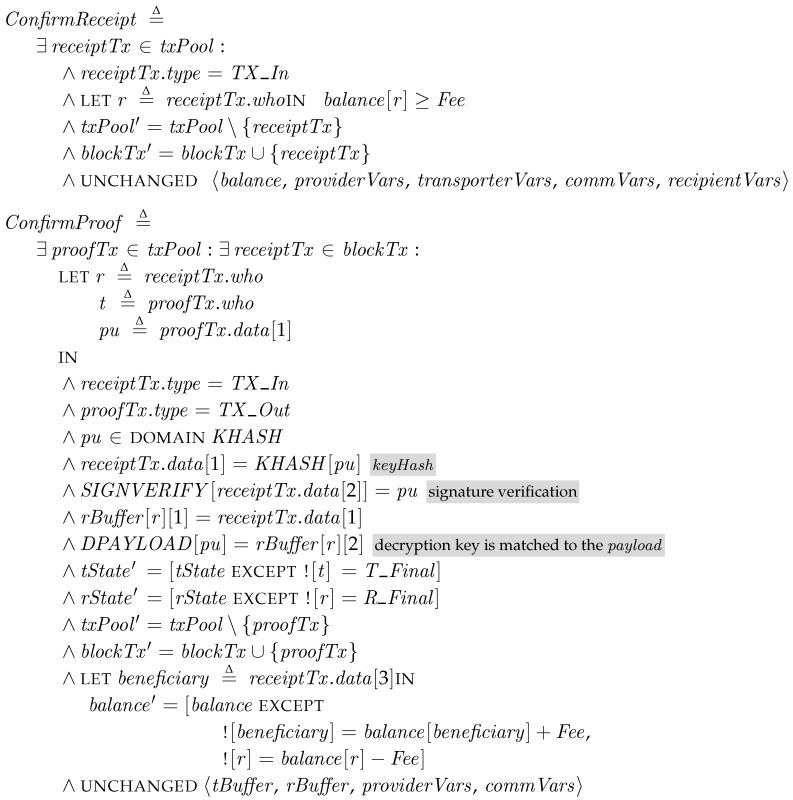


*Termination*. If all the recipients get the patch packages or all the patch packages are delivered, and the whole process is terminated. *Termination* has no primed variables since no more changes are required. Additionally, the conditions for the Faithful Delivery and Authenticated Origin properties are checked at the end of process. In Section 6.1, we more explain more about the conditions to check the properties.


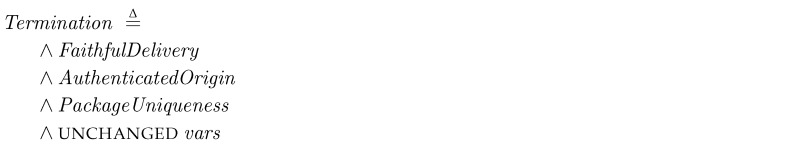


## 6. Analysis and Evaluation

In this section, we focus on answering the following questions.

- Is the proposed approach working correctly (with respect to the correctness properties)?
- Can all the participants get the proper rewards after the whole process?
- Can this method be implemented on the top of an existing blockchain system?

The first question is to check the correctness of the system. The second question is about whether all the participants can achieve their own goals. In particular, these are important questions for the incentive-based method of self-interested entities. It is hard to ensure the correctness of a decentralized system due to numerous, complicated combinations of participants’ states. Thus, we employ the model checking method to answer the first two questions. We run TLC the model checker for TLA^+^ on the specification of PatchTransporter in Section 5. However, the model checking can check every possible combinations of participants’ behaviors, but it is hard to check every possible attack since all the unexpected behaviors of attackers should be described in the model. Therefore, we simulated our system by implementing it on the Ethereum blockchain system to answer the last question and tested the result of the identified attacks in Section 3 with it.

### 6.1. Model Checking with Correctness Properties

The TLC model checker traverses all possible states and finds unexpected behaviors, e.g., deadlocks, and violation of invariant conditions. We represent the properties of Section 3 as the conditions for the specification. The properties are checked at the last action, *Termination*, or being checked at every possible state (Note that we regard safety conditions and invariants together as properties here.).

*Faithful Delivery and Authenticated Origin*. The transporters should deliver the authenticated patch package faithfully. The properties for Faithful Delivery and Authentication Origin are represented in the following conditions:


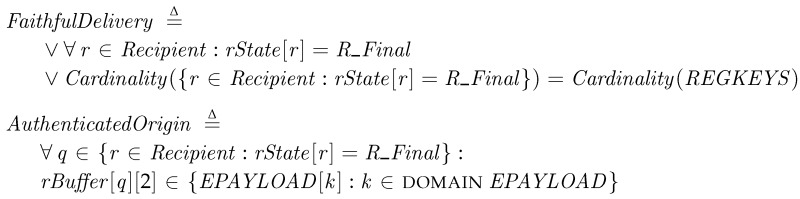


If the transporters have correctly delivered all the patch packages, all the recipients that have received a package must reach the final state, *R_Final*. Thus, we check that the number of the prepared packages, Cardinality(REGKEYS), is the same as the number of the recipient at the final state. In addition, we check that the payload of the received package is exactly the same as the original copy of the package in the provider, *EPAYLOAD*, to authenticate the origin and the message. These conditions are checked at the final step, *Termination*.

*Fair Reward*. To check the Fair Reward property, we inspect the different aspects of the property in the following conditions. First, we check that every transporter can get a reward as much as it contributes. For the fairness, every transporter delivers a unique package to a recipient and no transporter must get doubly paid for a single delivery. *PackageUniqueness* is checked at *Termination* and means that every recipient has a unique package in its buffer, where *rBuffer*[*r*][1] is the key hash of the received package and a set {·} in TLA^+^ contains unique elements only. We then check no transporter get doubly paid for a single package. The invariant condition *NoDoubleIncome* asserts no confirmed transaction doubly pays for the same key at all time.


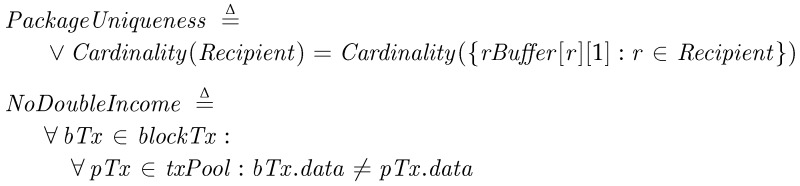


We also investigate every payment of the fee is properly carried out. The *NoUnpaidFee* condition is true if the balance of every recipients at the final state is decreased by the given *Fee*. *TotalBalanceInvariance* is a condition to check the total sum of the balances of all participants is not changed. Since the both are the invariants in PatchTransporter, we let them be checked at every state.


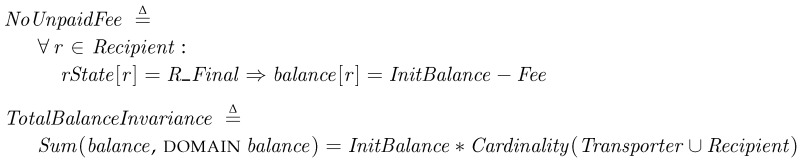


*Model checking results and validation*. We employ the model checking for mainly two purposes. First, we ran TLC to debug our specification. We could find errors in specification and revise them. The most errors encountered in developing the specification were related to progressing states. When the specification does not fully describe the behavior of the proposed system, an error occurs since TLC cannot find any proper next state. As adding more entities, i.e., multiple transporters and recipients, we could find more deadlock cases in our specification and then revise our protocol repeatedly. For example, when multiple transporters deliver packages to the same recipient, the system gets unexpectedly halted in some cases if all the packages are not properly handled with explicitly sending replies. Due to the model checking, we could revise our protocol to work correctly. The second purpose of using TLC is to check the properties. By adding all the safety properties and the invariants to the specification, we check the proposed model can correctly operate in all possible cases.  Table 1 shows the model checking results with the properties on the macOS High Sierra 10.13 with 3.1-GHz Intel i5 dual-core CPU (ModelID: MacBookPro14,2). With four transporters and three recipients, the whole model checking process took 30 s and visited 1,010,776 distinct states. After revising our specification, we got no errors with the correctness properties. In other words, if a system correctly implements the specification, it will work as we expect.

### 6.2. Simulation and Implementation

We implemented PatchTransporter in Python with the Ethereum system. Since we propose a new incentivized system to effectively deliver the patch packages, we focus on operability and security (The scalability and the delay in the confirmation phases depends on the blockchain system.). Hence, the goals of our simulation are two-fold: (1) to check whether the overall system can properly work with real operations, in particular, cryptographic operation and blockchain smart contracts and (2) to check whether the correctness properties in Section 6.1 are not violated in the implementation.

#### 6.2.1. Implementation and Performance

We implemented the behaviors of the main participants, the transporters and the recipient, in Python based on the specification in Section 5 and built a private Ethereum blockchain system for the blockchain part. In a holistic view, the participants are simulated with the real blockchain system. To inter-operate the Python objects and the blockchain system, we used web3py (Web3 Ethereum Interface in Python [55]), which is a Python implementation of the web3 interface in Ethereum (Web3 Ethereum Javascript Interface. [56]).

The receipt is implemented as a smart contract written in the Solidity programming language as shown in Algorithm 1 in Section 4. It is deployed by setting the key hash of the decryption key and the address of beneficiary, i.e., the transporter. After the deployment, the registered transporter who can reveal the decryption key by calling proofOfKey gets transferred the fee holding in the receipt.

We also implement the receipt validation in Section 4.4 as shown in Algorithm 2. First, we disassemble the bytecode of the target receipt into EVM assembly by using the evm tool from geth [57]. For the structure validation, we check whether the target code has the method selector for the function proofOfKey, i.e., PUSH4 0x9086f6fc … JUMPI. In addition, then we extract the instructions of proofOfKey. For the reference function, we compile the smart contract code of the receipt with Solidity 0.4.19 with optimization and extract the instructions in the same way.

The simulations ran with 150 recipients and 40 transporters. Through the simulation, we can observe that the most time-consuming part is the confirmation time in the blockchain system. The whole process requires two sequential confirmations: one for receipt and the other for key disclosure (redemption). That is, it takes at least two blocktimes, each of which is of the order of magnitude of seconds (The blocktime is larger than 13 s as of December, 2017, according to Ethereum Average BlockTime Chart [58]). In our simulation, to efficiently scale up the number of recipients and transporters in the private chain, we leveraged the blocktime by modifying the Ethereum source code to fix the block difficulty. (However, it is expected that ongoing projects on scaling up the blockchain system [59] will reduce the performance limitations in near future.) Since the blocktime for the confirmations is the most dominant performance overhead in the simulation, we focus more on checking the conditions of Section 6.1 and security of PatchTransporter in this section.

#### 6.2.2. Properties

*Faithful Delivery and Authenticated Origin*. Their properties check whether every recipient receives a valid patch package. The validity of the patch package is determined by the cryptographic operations. We use the PyCrypto library for the purpose. In the simulation, we use the Key Registration method, where a transporter registers a pair of its public key and the hash of private key to the provider. Thus, each patch package is doubly encrypted by a preshared key *s* and the registered public key. Since *s* is shared only between the recipient and the provider, the origin of the package is authenticated by successfully decrypting the payload of the package. We checked the conditions for the properties in Section 6.1 in the simulation and observed that all the recipients can successfully decrypt the delivered patch package.

*Fair reward*. To check the reward fairness, we set a malicious transporter. It makes multiple copies of a single patch package and delivers the copies to multiple recipients. Due to the characteristics of the blockchain, the recipient can find that there are different receipts of the same condition by investigating the publicly revealed information. Thus, once the key for one of the receipts is revealed, the other recipient can learn the decryption key without generating any receipt. In this way, we can see that the violation of *PackageUniqueness* is detected and *NoDoubleIncome* is satisfied.

The blockchain system also plays its role in the enforcement of *NoUnpaidFee* and *TotalBalanceInvariance*. We checked the balances of the recipients during the simulation. In the blockchain system, the miners keeps verifying the execution of the receipt smart contract code; thus, we could not observe any mismatching cases. Thus, we can verify that the conditions of *NoUnpaidFee* and *TotalBalanceInvariance* are satisfied.

#### 6.2.3. Attacks

We also check the effect of the possible attacks on PatchTransporter through the simulation.

*Package modification*. The modification on the patch package can be done on the patch payload or metadata. In simulation, we assume the modification attack and flipped a random byte of patch payload and metadata in the transporter, but every change is correctly detected. In particular, with the Key Registration method, since no participant cannot fully control the keys, any unnoticeable modification on the payload is not possible.

*Malformed Receipt*. The malformed receipt is detected by the receipt validation (Section 4.4). As we described in Section 6.2.1, the transporter first preforms the structure validation. If it cannot find the redemption function, proofOfKey, the receipt is rejected. Otherwise, it can have the instruction trace of the target code. By comparing the trace with that of the reference code, the transporter can validate it. Even if the two traces are not exactly matched, it checks whether there are the unexpected revert or gas-costly instructions in the target receipt. Thus, we set a malicious recipient, who randomly adds revert (or invalid) or gas-costly instructions when creating the receipt. Obviously, with the exact matching, every attack can be detected. We could also observe that redundant gas-costly instructions are easily detected since the EVM assembly is relatively simple and the straightforward disassemble process produces correct results. In particular, since the number of gas-costly instructions is not changed with and without optimization in Solidity 0.4.19, we could use the minimum threshold, i.e., 1, for $TH_{G}$ in detecting the gas-costly instructions. However, the length of paths to check unexpected revert is dependent on the optimization. The maximum different of path lengths is 13 between the optimized and unoptimized code. Thus, the threshold for the path length, $TH_{L}$, should be higher than 13 to prevent false positives caused by the optimization options from the same code. The detection rate of unexpected revert is higher as $TH_{L}$ is lower, but when $TH_{L}=13$, we can get 75.7% and 78.7% for optimized and unoptimized code. The limitation of the receipt validation can be improved by complicated analysis approaches, such as symbolic execution [24]. However, since we assume it is performed on the transporter for every receipt, the trade-off between efficiency and correctness should be considered.

*Fee Interception and Transporter Outcast*. Since we add the beneficiary of the fee as an argument of the constructor of the receipt, the fee can be transferred to the registered beneficiary only. Thus, even though an attacker replays the key disclosure at the same block time, the beneficiary is the same. Due to the deployed smart contract is immutable, we can verify that any attempt to change is not possible, including the owner change attack in the Parity Multisig Wallet Hack. In addition, when we use the Key Registration method, the encrypted payload can be decrypted by the private key, but only the transporter has it. Therefore, the provider and the recipient cannot collaboratively deceive the transporter for free delivery.

*Denial of Service Attack on Recipients*. A malicious transporter can forge a patch package of a legitimate format to consume the energy of the recipient while processing it. In our approach, the major energy consumption in the WSN/IoT recipient happens in creating a receipt and decrypting package, which require public key cryptography operations (when the Key Pool method is used, the cost for the decryption can be reduced to that of symmetric encryption.). Thus, the one of the attackers’ goals is to deceive the recipient to create a false receipt. However, the forged package can be detected in validating the metadata in PatchTransporter. Additionally, even if the malicious transporter employs the hash value of a wrong key as described in Section 3, it cannot pass the validation test in the receipt smart contract. Thus, the recipient can know that the revealed key is not correct and skip the unnecessary decryption with the wrong key. In this way, the energy-expensive operation is avoidable in PatchTransporter.

## 7. Related Work

*Software dissemination*. The methods for secure software delivery are researched in WSN areas. Dutta et al. [4] presented Deluge to securely deliver software updates in WSNs. One of the characteristics of wireless communication is the lossy channel. Deluge handles the lossy wireless channel to present reliable software update dissemination. The secure software delivery is also crucial in the WSN and IoT environments. Liu et al. [6] proposed dynamically updated software in various configurations of sinks and sensors. They focused on effective dissemination of software updates in WSN and IoT environments. Hong et al. [5] presented a method to reprogram IoT devices by sending binary objects from smart phones. The smartphone composes a new software for IoT device and delivers it. They utilize the enhanced capacity of current smartphones and the communication ability of IoT devices to deliver and update new software. These methods treat the payload of software update like a binary data. However, Lee et al. proposed CodeDog [7] and employed a specific markers to find semantic modification in a fragmented program code from a sequence of packets, gathered by network monitors. Cheng et al. rather focused on prioritizing nodes to patch first. A node handling more traffic is more important in propagation of attacks. The scheme updates the critical points first to improve the health of a whole network. Most of the previous work improves the reliability of the delivery but do not care about the discovery of patch. Even hardening the delivery process, the effectiveness of the patch closely depends on the rapidness of patch deployment. Our approach is to construct a new ecosystem of distributed workers. We try to enhance the software update process in a different view. We let the recipient device actively participate in the software update process and transform a software update process to a profitable service for self-interested patch service providers. As the number of devices increases due to the advances in WSN and IoT, the decentralized approach like ours is advantageous with respect to scalability and maintenance cost.

*Blockchain on WSN/IoT*. Blockchain in the WSN and IoT environments helps inter-operation among devices and offloads the resource-expensive cryptography operations. Based on the improvement in identity and interoperability, various applications of blockchain and IoT are introduced. The blockchain secures payment through IoT devices and enables micro-payments with the cryptocurrency systems. For example, sharing the real properties, such as house and cars, is automated by the smart contract, and complicated international shipment monitoring is also secured by combining the blockchain and WSN/IoT [43]. Applying the blockchain to enhance security of existing methods is also actively being tried. In particular, Boudguiga et al. [60] proposed an IoT update method using blockchain, focusing on securing the patch. The update availability is checked on the blockchain and the recipient-initiated update process also checks the innocuousness of the patches in a distributed manner like majority voting of devices. However, PatchTransporter has a mediator-initiated push approach. In particular, we accelerate the patch delivery by encouraging the competition among the transporters, which is important to effectively reducing the damage from cyber attacks.

*Monetizing delegations*. Before the crypto-currencies, the payment for the service remained in the field of financial institutes and the service itself resided on the data networks. However the blockchain system and the crypto-currencies integrate the financial flows into data networks. We could observe that any work can be outsourced in previous services like Amazon Mechanical Turk [19]. The blockchain system transforms collected data or automated processes into payable services and various approaches are being tried [61]. In this sense, we convert the controlled patch service to the delegated service by decentralized workers.

*Blockchain and fair exchange*. Traditionally, the fair exchange has been researched with cryptographic operations [20,21]. However, recently, it is extended to the escrow service with cryptocurrency and blockchain systems. Goldfeder et al. [23] presented the escrow service based on Bitcoin. Due to the irreversible transactions of cryptocurrency, they employed Bitcoin Script in enforcing the rules with additional cryptographic operations, e.g., threshold cryptography, to prevent unfair transactions in advance. Juels et al. [22] proposed the secure exchange of funds and secrets by using Ethereum smart contracts. These works are closely related to our proposed scheme, but their applications focus on reliable escrow and public leakage. In addition, both do not consider the validation of the malformed smart contract code. In the context of applying patches to WSN/IoT devices, the proposed scheme provides secure and practical delivery system in exchanging fee and the patch package with efficiently validating the receipts and packages.

*Blockchain Security*. Even though the blockchain system provides a reliable platform in the distributed manner, the security of blockchain is being actively analyzed with respect to various aspects. Luu et al. [24], Halpin et al. [25] and Li et al. [26] systematically surveyed the vulnerabilities of blockchain consensus, smart contracts and attacks. In particular, to check the existence of known security vulnerabilities, Luu et al. proposed Oyente, the symbolic executor on the EVM bytecode [24]. It follows the control flow and continuously checks the conditions of vulnerabilities, such as transaction ordering dependence, mishandled exceptions, and reentrancy. Focusing more on the security of the smart contract, Atzei et al. [27] analyzed the attacks on the smart contracts. In the EVM execution environment, the smart contract code has several vulnerable programming patterns: e.g., incompatible type cast and gasless sent, and immutable bugs. Chen et al. [28] investigated the gas-consumption specific problems and identifies seven gas-costly patterns that cause uselessly spent gas. Based on the related work, we analyze the possible vulnerabilities and attacks on managing the receipt smart contracts, and propose the countermeasures such as the receipt validation.

## 8. Conclusions

In the complicated WSN and IoT environments, securely delivering software patches is a hard and critical problem. In this paper, we propose a new approach, PatchTransporter, for delivering patches through an incentive system. To ensure that the reward system is trustworthy, we employ the blockchain system as an enforcer of commitments between a patch transporter and a recipient device. We also propose the self-checking process to protect the participants from the domain-specific vulnerabilities in the blockchain reward system. Furthermore, we develop the specification to clearly describe the proposed system. In particular, on the correctness properties, the model checking with the specification and the simulation with the real blockchain system show that the proposed system can provide proper incentive systems in decentralized approaches.

## Figures and Tables

**Table 1:** Model checking results of PatchTransporter with four transporters and three recipients.

Time	Depth	States Found	Distinct States	Errors
30”	28	3,164,524	1,010,776	0

## Appendix A

The following shows the complete specification of PatchTransporter.
